# KIM-1, IL-18, and NGAL, in the Machine Learning Prediction of Kidney Injury among Children Undergoing Hematopoietic Stem Cell Transplantation—A Pilot Study

**DOI:** 10.3390/ijms242115791

**Published:** 2023-10-31

**Authors:** Kinga Musiał, Jakub Stojanowski, Justyna Miśkiewicz-Bujna, Krzysztof Kałwak, Marek Ussowicz

**Affiliations:** 1 Department of Pediatric Nephrology, Wrocław Medical University, Borowska 213, 50-556 Wrocław, Poland; 2 Department of Nephrology and Transplantation Medicine, Wrocław Medical University, 50-556 Wrocław, Poland; 3 Clinical Department of Pediatric Oncology and Hematology, Mother and Child Health Center, Karol Marcinkowski University Hospital, 65-046 Zielona Góra, Poland; 4 Department of Pediatric Bone Marrow Transplantation, Oncology and Hematology, Wrocław Medical University, 50-556 Wrocław, Poland

**Keywords:** acute kidney disease, acute kidney injury, allogeneic hematopoietic stem cell transplantation, augmented renal clearance, hyperfiltration, random forest classifier, tubular damage

## Abstract

Children undergoing allogeneic hematopoietic stem cell transplantation (HSCT) are prone to developing acute kidney injury (AKI). Markers of kidney damage: kidney injury molecule (KIM)-1, interleukin (IL)-18, and neutrophil gelatinase-associated lipocalin (NGAL) may ease early diagnosis of AKI. The aim of this study was to assess serum concentrations of KIM-1, IL-18, and NGAL in children undergoing HSCT in relation to classical markers of kidney function (creatinine, cystatin C, estimated glomerular filtration rate (eGFR)) and to analyze their usefulness as predictors of kidney damage with the use of artificial intelligence tools. Serum concentrations of KIM-1, IL-18, NGAL, and cystatin C were assessed by ELISA in 27 children undergoing HSCT before transplantation and up to 4 weeks after the procedure. The data was used to build a Random Forest Classifier (RFC) model of renal injury prediction. The RFC model established on the basis of 3 input variables, KIM-1, IL-18, and NGAL concentrations in the serum of children before HSCT, was able to effectively assess the rate of patients with hyperfiltration, a surrogate marker of kidney injury 4 weeks after the procedure. With the use of the RFC model, serum KIM-1, IL-18, and NGAL may serve as markers of incipient renal dysfunction in children after HSCT.

## 1. Introduction

Children undergoing allogeneic hematopoietic stem cell transplantation (HSCT) are vulnerable to acute kidney injury (AKI), especially in the early post-transplantation period [1,2,3,4]. The major risk factors of AKI development are matched unrelated donation, cord blood stem cell transplantation, and veno-occlusive disease/sinusoidal obstruction syndrome (VOD/SOS) [5,6]. Moreover, aggressive immunosuppression and infectious complications increase the incidence of AKI [7,8]. Therefore, the early identification of patients at risk of developing kidney damage may enable the modifications in therapeutic decisions, with subsequent prevention, or at least reduction, of AKI occurrence. The fast identification of AKI features is also essential for the prognosis of kidney function—the earlier the diagnosis, the more probable the complete reversal of renal impairment. 

The increased eGFR values are also associated with the higher risk of recurrent AKI and subsequent development of acute kidney disease (AKD) in the pediatric population undergoing HSCT [9]. This paradox may be partly explained by the fact that patients undergoing HSCT due to oncological reasons may present with hyperfiltration already before the transplantation [10]. The latter could be the consequence of a gradual decrease in the nephron mass, owing to the nephrotoxicity of chemotherapeutics and subsequent compensative hyperfiltration concerning the remaining nephrons [11]. Therefore, chronic hyperfiltration may indicate incipient kidney dysfunction, as it was confirmed in cancer survivors [12]. 

In the meantime, the malnutrition and hypermetabolic state of the patient, together with the routine intensive hydration during the procedure and through the first 3 weeks after HSCT, as well as subsequent forced diuresis, may diminish the serum creatinine concentration, thus increasing the value of estimated glomerular filtration rate (eGFR) [13]. Moreover, the eGFR value may serve as a reliable marker of kidney function only under stable metabolic and hemodynamic conditions.

Consequently, the potential underrating of the serum creatinine concentration and overrating of the eGFR values make the assessment of the degree of kidney damage during the first month after HSCT a challenge. Thus, a new classification of AKI, taking into account both classical parameters of kidney function (serum creatinine or eGFR) and the indices of cell damage, seems a more appropriate tool in the assessment of kidney injury after HSCT. The category of subclinical AKI was defined as an intact kidney function in the presence of positive damage markers [14,15,16]. The combination of functional and damage markers has increased the efficiency of AKI diagnosis in patients after cardiosurgery [17]. The preliminary reports also show its effectiveness in the children after HSCT [18,19]. Consequently, the definition of AKD has also been expanded, taking into account the renal damage and the loss of renal reserve as markers of both early and prolonged kidney dysfunction (Table 1).

The markers of tubular dysfunction and damage, like kidney injury molecule (KIM)-1, neutrophil gelatinase-associated lipocalin (NGAL), or interleukin (IL)-18, are better indices of early renal injury than serum creatinine, so they may be of added value while diagnosing the severity of AKI in the HSCT population [20,21,22]. However, their ability to predict renal dysfunction has not been proven yet [23]. Artificial intelligence is a new tool in the analysis of clinical data, able to create predictive models based on the retrospectively collected data [24]. It has been used for AKI prediction in patients after cardiac surgery or those hospitalized in intensive care units but not in patients undergoing HSCT–neither children nor adults [25,26]. 

## 2. Aim of Study

The aim of this study was to assess the serum concentrations of damage markers (KIM-1, IL-18, NGAL) in children undergoing HSCT and confront them with the classical functional markers (creatinine, cystatin C), and hyperfiltration being the surrogate marker of renal dysfunction. Another goal was to analyze the potential value of serum KIM-1, IL-18, and NGAL concentrations as predictors of kidney damage in children after alloHSCT with the use of artificial intelligence tools. 

## 3. Results

### 3.1. Serum KIM-1, IL-18, NGAL, Cystatin C, Creatinine, eGFR

**Figure 1:**
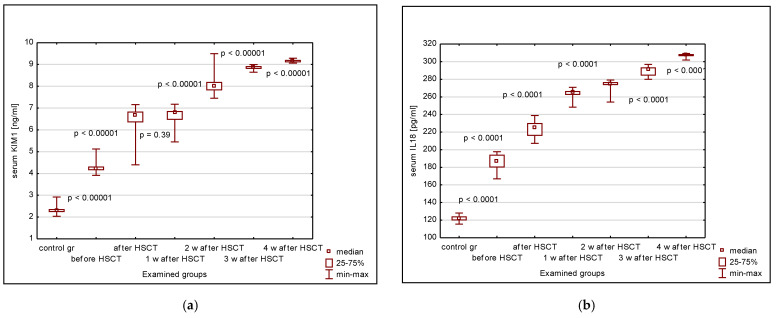

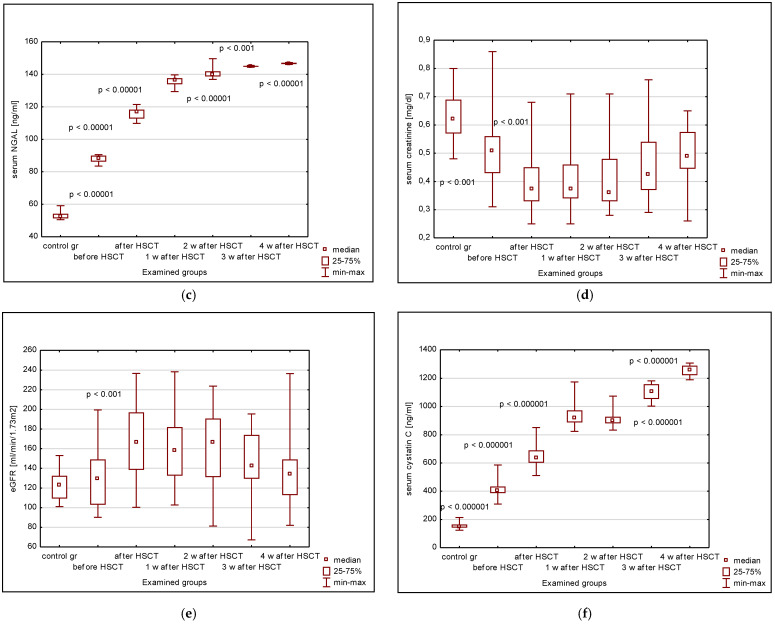
Serum concentrations of examined damage markers, parameters of kidney function, and indices of glomerular filtration in controls and HSCT patients before transplantation and until 4 weeks after the procedure: (**a**) KIM-1; (**b**) IL-18; (**c**) NGAL; (**d**) creatinine; (**e**) estimated glomerular filtration rate (eGFR); (**f**) cystatin C; KIM-1–kidney injury molecule-1; IL-18–interleukin-18; NGAL–neutrophil gelatinase-associated lipocalin; HSCT–hematopoietic stem cell transplantation; 1 w–one week; 2 w–two weeks; 3 w–three weeks; 4 w–four weeks.

The serum KIM-1, IL-18, and NGAL concentrations were significantly higher in the children undergoing HSCT versus the control group, irrespective of the time period since transplantation (Figure 1a–c). Additionally, the values in the study group have increased systematically until the fourth week after HSCT, with statistically significant differences between the subsequent observation points (Figure 1a–c). The serum creatinine values were decreased in the HSCT patients versus controls at any time point after HSCT, with their nadir between the first and the second week after HSCT (Figure 1d). Four weeks after HSCT, the serum concentrations of KIM-1, IL-18, and NGAL were comparable to those before transplantation. 

Contrarily, the median eGFR values before transplantation did not differ from those in the control group (Figure 1e). A significant increase was observed just after HSCT, followed by fluctuations until the third week and a return to the pre-transplantation values after 4 weeks (Figure 1e). However, the variability of eGFR values in the studied group, significant already before HSCT, has even increased 4 weeks after the procedure, as has the rate of patients with hyperfiltration. The fluctuations of serum cystatin C (cys C) concentrations were similar to those seen in the case of serum KIM-1, IL-18, and NGAL (Figure 1f). 

Four patients developed AKI (Risk–3, Injury-1), and none of the episodes occurred in the fourth week after transplantation.

### 3.2. The Random Forest Classifier (RFC) Model

The RFC model [27] was set up based on the clinical data (age, gender, BMI), the basic laboratory results, including serum creatinine, eGFR values, serum cystatin C, and the analyzed damage markers (KIM-1, IL-18, and NGAL). All input variables were assessed in the fixed time points–before HSCT, 24 h after transplantation, and then 1, 2, 3, and 4 weeks after the procedure. The subsequent observations were completed in 22 patients and served as the database for establishing RFC. 

The recursively selected subsets of variables constituted the input of a random forest classifier (RFC). Each variant has been optimized, taking into account accuracy, AUROC, precision, recall, and MCC. Then, the best model was saved. The GINI importance was measured to define the parameter with the largest share in the prediction.

**Figure 2:**
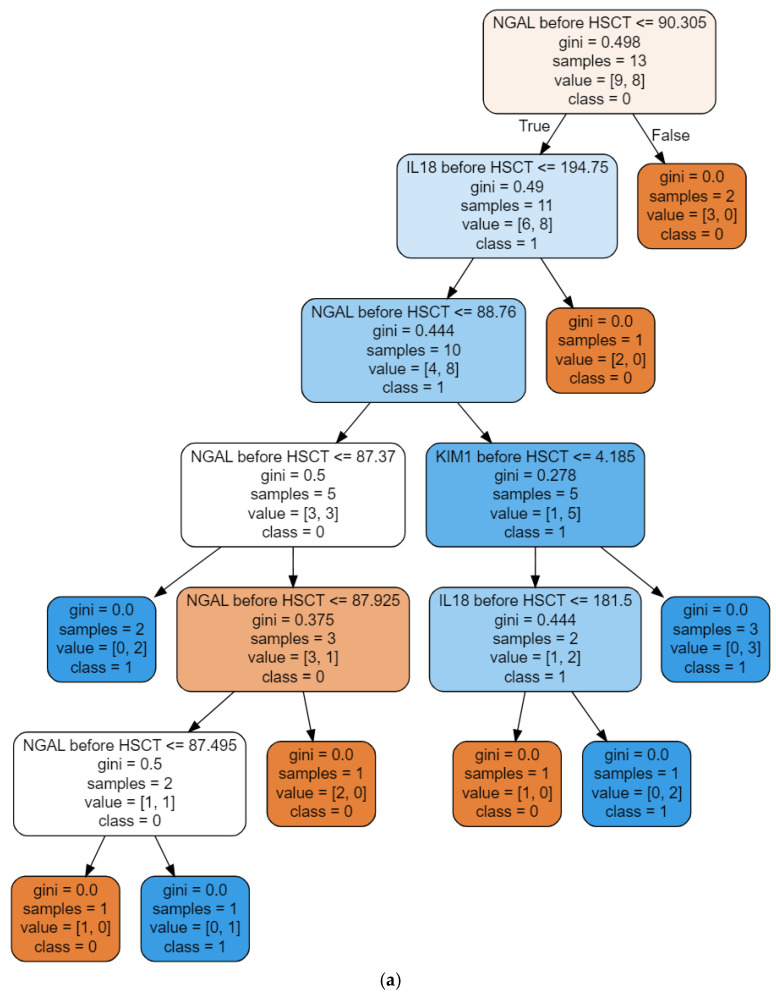

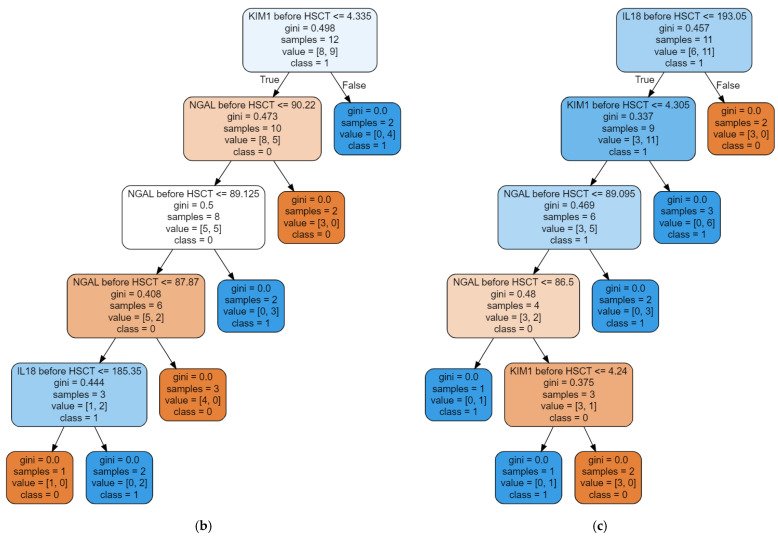
The selected decision trees from the RFC model, with serum NGAL (**a**), KIM-1 (**b**), and IL-18 (**c**) as input variables, predicted hyperfiltration 4 weeks after alloHSCT.

Finally, the model built on the basis of 3 input variables (the serum concentrations of KIM-1, IL-18, and NGAL in children before HSCT) was able to assess the rate of patients with hyperfiltration 4 weeks after the procedure in the most accurate way (the exemplary decision trees are shown in Figure 2).

**Figure 3:**
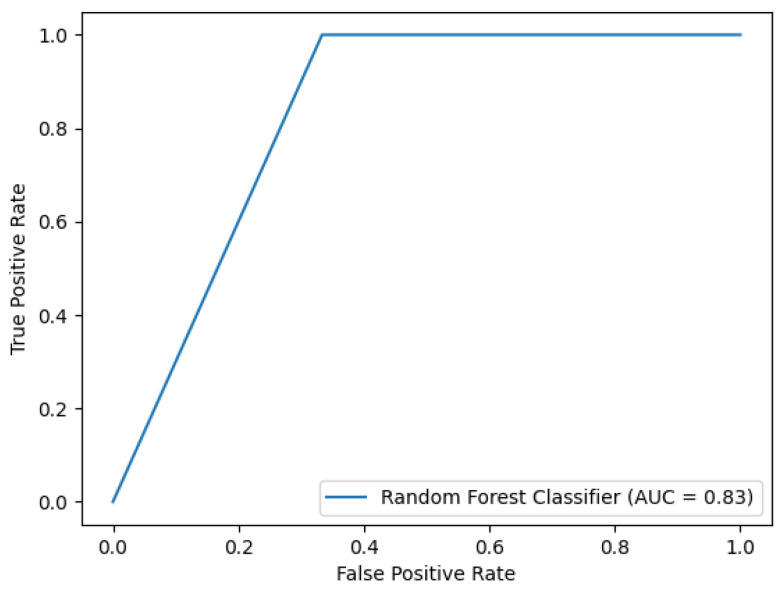
ROC for the RFC model, containing KIM-1, IL-18, and NGAL serum concentrations before HSCT as input variables.

The RF Classifier achieved the AUROC of 0.8333, accuracy of 80.00%, positive predictive value of 0.8667, and sensitivity of 0.8000 (Figure 3). The contributions of KIM-1, IL-18, and NGAL to the prediction in this model were comparable (33.73%, 32.77%, and 33.5%, respectively).

## 4. Discussion

With its pooled incidence nearing 50%, AKI remains a common condition among children undergoing HSCT [5]. The AKI incidence based on the pRIFLE criteria overrates that diagnosed with the KDIGO classification [28]. Therefore, eGFR may seem more sensitive than serum creatinine as a tool for the early assessment of kidney function decline. In our group, the AKI incidence was as low as 15%, which might refer to the short observation period and the protective scenarios implemented within the first 3 weeks after HSCT. 

In detail, owing to the fact that the patients are specifically prone to AKI in the early post-transplantation period, the clearly defined interventions (intensive hydration in order to protect against hypoperfusion and drug toxicity, followed by the forced diuresis) proceeded to minimize the risk of kidney injury. However, the above-mentioned protocols result in increased renal preload and augmented renal clearance. These modifications seem to disqualify the serum creatinine and eGFR from being reliable markers of renal function, mainly owing to the instability of their values over time. Apart from the bias related to the hydration status, the serum creatinine increase appears later than that of the damage markers, so its predictive ability may be insufficient under time pressure. 

These conditions have emerged in the need for alternative indices in AKI diagnostics. The proposed new definition of AKI has emphasized the rise of damage markers preceding functional renal impairment [23]. Indeed, stage 1S has introduced the phenomenon of isolated biomarker increase in the absence of serum creatinine elevation or eGFR decrease [23]. A plethora of markers have been tested so far, and many of them have revealed usefulness in the early identification of subclinical AKI [29,30]. Among them, NGAL, KIM-1, and IL-18 were most often studied in patients with AKI and gave the most promising results regarding the early diagnosis and the stratification of AKI risk and outcome [20,21,22,31,32,33,34]. Despite these convincing data, the spectrum of markers assessed in the patients undergoing HSCT is still narrow, and only a few studies have dealt with this subject in the pediatric population.

Benoit et al. [18] have broadened the spectrum of biomarkers used in the classification of the severity of AKI in children after HSCT. First, they implemented the patients with solitary urinary NGAL elevation. Second, they stratified the severity of AKI by distinguishing between the mild and severe elevation of uNGAL, together with the assessment of serum creatinine or cystatin C-based eGFR criteria [18]. Our previous investigation identified the urinary clusterin as a candidate early marker of renal damage in children undergoing HSCT, outperforming the urinary KIM-1 and cystatin C [19].

Our current results have revealed the universality of the damage biomarker positivity in the children undergoing HSCT and proved that any pediatric patient planned for the transplantation may present with the features of subclinical kidney damage. Moreover, it became clear that serum creatinine and the creatinine-based eGFR are insufficient tools for the adequate evaluation of glomerular filtration. This discrepancy was most evident during the first 2 weeks after transplantation when the serum creatinine concentrations achieved their nadir, and the eGFR values reached zenith. On the other hand, serum cystatin C has demonstrated systematically increasing concentrations throughout the early post-transplantation period. As the cystatin C values are independent of the muscle mass, they proved their superiority over the serum creatinine in the assessment of kidney function in HSCT children. 

However, the scale of the divergence between the serum creatinine/eGFR, returning to the pre-HSCT values after transplantation, and serum cystatin C, maintaining the increasing trend in the biomarker activity until the fourth week of observation, deserves more attention. The possible explanation of such variance may, at least partly, lie in the fact that the glomeruli freely filter cystatin C which is not secreted by the tubules, contrarily to creatinine and other damage markers assessed in our study. Therefore, it may serve as a more reliable marker of glomerular filtration. 

Indeed, the cystatin C-based eGFR values would have made the analysis even more precise, but in our study group, the assessment of cystatin C did not belong to the routine hematological protocol, so the only option was to evaluate its concentration for the purpose of research. Unfortunately, such results cannot be implemented into the diagnostic calculation of the serum creatinine and cystatin C-based eGFR. However, Benoit et al. [18] have implemented serum cystatin C and cystatin C-based eGFR into the renal function assessment. And yet, even the cystatin C-related modifications were unable to eliminate the hyperfiltration, although its incidence has decreased. 

Our goal was to compensate for this shortcoming with the complex analysis of damage markers, taking into account their multi-functionality. Therefore, we chose the indices of known affinity to certain chemotherapeutics in order to assess the mechanisms of toxic and hemodynamic interactions. Cystatin C reflected the impact of chemotherapy on the kidney [35]. The recent meta-analysis revealed that cys C is a promising candidate for the assessment of early kidney dysfunction in patients undergoing chemotherapy, outperforming the serum creatinine decrease, similar to our observations [35]. The potential for the diagnosis of drug toxicity is also characteristic of NGAL, which is sensitive to AKI induced by calcineurin inhibitors [36]. The murine and rat models of cisplatin-induced AKI proved sNGAL accuracy in predicting early tubular damage, although late prognosis could not be tested due to high animal mortality by the sixth day of the experiment [37]. However, sNGAL turned out to be a sensitive marker of LPS-stimulation and immune-mediated glomerular damage, which, in the light of specific, HSCT-driven inflammatory, toxic, and immune conditions, suggested its multifunctional usefulness in the prognosis of future renal damage [37].

Indeed, such pluripotency of the examined markers could, at least partly, justify the permanent increase of their concentrations, in contrast with the creatinine and eGFR values, returning to the pre-transplantation records after 4 weeks. The toxic effect of chemotherapy could add to vasoconstriction, thus decreasing glomerular filtration, as in the case of cyclosporin use. Thus, the cumulative effect of multilayer destructive mechanisms could explain the successive rise of NGAL, IL-18, and KIM-1 concentrations until the end of the observation period. 

However, these results did not solve the puzzle of the potential reversibility of the observed elevation of serum NGAL, KIM-1, IL-18, and cystatin C concentration. The biomarker increase has endured for 4 weeks, which should automatically suggest the diagnosis of acute kidney disease (AKD) instead of AKI [38]. Indeed, our patients fulfilled the AKD stage 0B criteria when serum creatinine values were normal, but the features of renal damage were present, and the renal reserve was lost. In detail, the increased biomarkers coexisted with normal or even decreased, serum creatinine concentrations. Meanwhile, the eGFR values peaked just after HSCT and then returned to pre-HSCT records after 4 weeks. Therefore, in light of the serum creatinine and eGFR-related bias, the routine use of cys C as a primary marker of glomerular filtration, is strongly suggested in the specific groups of patients, e.g., those after HSCT.

Of note, the hematological procedures for the fourth-week post-HSCT are already devoid of excessive hydration and diuretic use; thus, the eGFR values tested in this time point are stable, independent of any stimulation and more reliable than within the first 2–3 weeks after transplantation. Having said that, the hyperfiltration may suggest a prolonged period of kidney dysfunction, with the probability of developing acute kidney disease and further progression to chronic kidney disease (CKD). These conclusions are strengthened by the concomitant increased cys C concentrations, confirming the glomerular filtration impairment.

The ADQI guidelines consider hypothetical trajectories of the kidney function outcome after AKI [14]. However, out of 5 scenarios, only one concerns the normalization of kidney function, provided the improvement has started no later than 7 days after injury. On the contrary, the worse prognosis is due to the subacute AKD, with normal kidney function for at least 7 days, followed by the occult but steadily aggravating renal impairment. In the patients planned for HSCT, the undetected ongoing chronic kidney damage could create a functional overlap with the HSCT-related AKI/AKD, facilitating or even aggravating the further progression of subacute renal dysfunction. From that point of view, the fourth week seemed to be both the optimal and the last time interval to be tested regarding the prognosis of AKD aggravation or CKD development. 

Our next step was to verify the predictive potential of AI tools against AKI/AKD development, with the conscience of the preliminary character of the research among HSCT children and the limited power of its conclusions. Despite the vast interest in machine learning as a tool for AKI prediction, there is an ongoing discussion about its role in everyday practice decision-making, keeping clinicians reluctant to what modern statistical tools may offer [39,40]. So far, in single reports, the authors have proven the AI applicability in the prediction of renal replacement therapy in AKI or even have predicted future AKI in a continuous way [41,42]. However, pediatric research on the subject did not go beyond critical care conditions [43]. One of the potential contraindications could be the small study groups. However, the main aspect restricting the AI application is the difficulty of training a model. We managed to train it despite the small set of training data, and we were able to confirm its effectiveness with the use of several measures to describe the predictive properties. Additionally, the random forest classifier (RFC) is a relatively simple model, useful even if the number of variables is limited. In addition, the examples of RFC implementation into small datasets in genomics encouraged our attempts [44,45,46]. However, it is obvious that the value of our analysis would profit from being verified on larger datasets.

This work is the first attempt in the literature to predict the risk of renal damage before HSCT with AI tools. Indeed, the current tendency towards finding the sensitive markers of AKI multiplies the spectrum of new indices, but so far, none of them has fulfilled the criteria of identifying the potential candidates for AKI before HSCT. Thus, one possible option is to revolutionize how the data are analyzed instead of searching for new biomarkers.

The analysis performed in our study is an example of a new trend in the statistical analysis. It broadens the opportunities of AKI prediction by combining the molecular markers of known pathophysiology, like NGAL or IL-18, with the new methods of data analysis from the artificial intelligence perspective. In light of the paucity of markers assessing the risk of AKI/AKD development before the damage has started, the creation of a predictive model with the data on the current renal function as input variables sounds promising. These tools have not been implemented into the analysis of a population undergoing HSCT, neither children nor adults. Our preliminary results suggest that RFC is one of the optional modalities to predict kidney dysfunction based on the damage markers. In our opinion, it deserves further attention and should be applied to larger datasets of HSCT patients.

Another argument for its utility is the clinical importance of kidney damage prediction in the HSCT population. The preemptive stratification of patients vulnerable to AKI gives promise of personalized medicine, with the therapy tailored to both current kidney function and future scenarios of its impairment. The damage markers seem ideal candidates for such easy screening, provided the point-of-care testing becomes vastly available. 

Finally, we have to acknowledge the limitations of our study. The clinical data were collected according to the hematological protocols; thus, not all nephrological aspects, e.g., urine output or cystatin C evaluation with a standardized laboratory technique, could be analyzed in depth. The use of serum creatinine formed a bias owing to the decreased muscle mass of the patients and modified the values of eGFR. The latter could also depend on the iatrogenic interventions, including hydration of the patients and the diuretic use. The studied group was relatively small and heterogeneous, restricting the power of calculations with AI tools and limiting the strength of conclusions. Moreover, not all confounders potentially responsible for renal impairment occurrence and progression were considered. These restraints urge the continuation of this study throughout a longer period and on a larger group of patients in order to verify whether pre-HSCT damage markers are able to predict AKI or acute injury exacerbating the ongoing CKD, as well as AKI progression into AKD or further CKD progression in children undergoing HSCT.

## 5. Materials and Methods

### 5.1. Study Design and Settings

The study group contained 27 children (15 girls, 12 boys; median age 4, 5 years, interquartile range 3, 1–8, 0 years) undergoing their first HSCT in the Department of Pediatric Bone Marrow Transplantation, Oncology and Hematology in 2018. The observation period started before introducing conditioning therapy; parameter examinations were performed 24 h, 1, 2, 3, and 4 weeks after HSCT. 

The exclusion criteria for the patients were autologous HSCT, re-transplantation, age below 2 years, and over 18 years. Seventeen patients were qualified for transplantation due to oncological reasons, and 10 underwent HSCT due to non-oncological indications (mainly severe aplastic anemia). In 79% of cases, the donor was unrelated, 18% were related, and 3% were haploidentical. 

The patients received either the myeloablative (fludarabine, treosulphan, and thiotepa, or busulphan, cyclophosphamide, and fludarabine) or non-myeloablative (cyclophosphamide, fludarabine) regimens. In the majority of patients, graft versus host disease (GvHD) prevention consisted of pretransplant anti-thymocyte globulin (ATG), posttransplant cyclosporine A, and methotrexate. Nineteen out of 27 patients developed GvHD. None of the patients died during the first 4 weeks after HSCT.

The control group consisted of 18 age-matched children (nine girls, nine boys; median age 7, 8 y, interquartile range 7, 0–9, 8 y) with monosymptomatic nocturnal enuresis and normal kidney function. 

### 5.2. Methods

In the control group, the blood samples from HSCT patients were drawn from central venous access from peripheral veins after an overnight fast. Samples were left to clot for 30 min, centrifuged at 4 °C, 1000× *g* for 15 min, and then serum was stored at −80 °C until analysis. 

The serum concentrations of KIM-1, IL-18, NGAL, and cystatin C, were evaluated by commercially available ELISA kits (KIM-1-EIAab, reagent kit E0785h; IL-18-R&D Systems, reagent kit DL180; NGAL-R&D Systems, reagent kit DLCN20; cystatin C R&D Systems, reagent kit DSCTC0). Standards and serum samples were transferred to 96 well microplates pre-coated with recombinant antibodies to human KIM-1, IL-18, NGAL, and cystatin C. Captured proteins were then detected using monoclonal antibodies against KIM-1, IL-18, NGAL, and cystatin C, conjugated to horseradish peroxidase. Next, the assay was developed with tetramethylbenzidine substrate, and a blue color was developed proportionately to the amount of captured protein. The addition of acid stop solution ended the color development and converted it to the endpoint yellow. The intensity of the latter was measured in a microplate reader at 450 nm, with the correction wavelength at 550/650 nm. Each sample was tested in duplicate, and the arithmetical mean was considered the final result. Measurements were performed according to the manufacturer’s instructions, and results were calculated by reference to standard curves. The intra-assay and inter-assay coefficients of variation (%CV) for examined parameters did not exceed 10%.

The assessment of kidney function was based on the hematological protocols evaluating the serum creatinine concentration at fixed time points. The serum chemistry parameters were measured using the automated routine diagnostic tests on the Beckman Coulter AU2700 analyzer. The serum concentrations of all parameters were measured before conditioning, 24 hours after allotransplantation, and then 1 week, 2 weeks, 3 weeks, and 4 weeks after HSCT. eGFR was calculated in all time points using the Schwartz formula [47]. The serum creatinine and eGFR changes were confronted with the pre-transplantation values. Hyperfiltration was defined as eGFR ≥ 140mL/min/1.73m^2^ [48,49]. Acute kidney injury was defined according to the pRIFLE criteria [28]. 

### 5.3. Statistical Analysis

The results were expressed as the median values and interquartile ranges. The null hypothesis of normality of the distribution of analyzed variables was rejected by the Shapiro–Wilk test. Thus, the analysis was performed with the use of nonparametric tests (Friedman, Wilcoxon, Kruskal–Wallis, Mann–Whitney U). The statistical analysis was performed using the package Statistica ver. 13.3 (StatSoft). A *p*-value < 0.05 was considered significant.

### 5.4. Building the Random Forest Classifier Model

The purpose of the classifier [27] is to technically divide a heterogeneous input set due to the labels assigned to the elements, but based on the values stored in the records, e.g., laboratory results. In other words, the goal is to find, based on the given values, the most likely label for those values. 

A single decision tree node splits a set based on a condition contained in that node. In this case, it is a minority relation. If any of the splits leads to a set of elements with one type of label, it is said that there is zero impurity, and no more splits are made at that point. It is expected to achieve less class impurity with each node. 

The randomness of a decision tree means that a random set of variables is selected from the input variables to generate a single tree. This way, the amount of data encoded in the tree is reduced, and overfitting to the training data and insensitivity to the testing data are avoided. The use of a set of decision trees allows for covering the training set and better performance. 

RFC is a classifier insensitive to the scaling of input variables. In addition, RFC is characterized by greater stability in relation to the number of input variables, which means that, for example, a redundant input variable does not have to interfere with the generation of a forest with good performance. It can then be easily eliminated, and the entire model is optimized for clinical use.

The Gini feature importance [50] is the sum of average impurity declines in the whole tree. The greater the Gini feature importance, the greater the contribution of a given variable to the clarification of sets, and the greater the number of significant divisions.

## 6. Conclusions

KIM-1, IL-18, and NGAL are useful in assessing subclinical kidney injury in children undergoing HSCT. Their serum concentrations before transplantation may also serve as markers of incipient renal dysfunction 4 weeks after the procedure. The serum cystatin C concentration outperforms serum creatinine in the reliable assessment of kidney function in HSCT children. 

The optimal tool for AKI diagnosis in this population should be the simultaneous assessment of functional and damaged kidney markers. The developed Random Forest Classifier model seems clinically useful in targeting patients who are at risk of sustained kidney dysfunction after HSCT. RFC seems to be a promising tool for the continuous assessment of renal damage risk in children, starting before transplantation. This approach should be tested on a larger group of patients and could increase effectiveness by a longer observation period.

## Figures and Tables

**Table 1:** Stages of AKI/AKD [14,15] with modifications.

Type of Renal Dysfunction	Endurance of Dysfunction	Stages of AKI/AKD	Serum Creatinine	Markers of Damage
AKI	≤7 days	Subclinical AKI	Normal value	Biomarker positive
		Stage 1	1.5-fold increase	Biomarker positive or Biomarker negative ±
		Stage 2	2-fold increase	
		Stage 3	3-fold increase	
AKD	>7 days, but <3 months	Stage 0A	Return to baseline values	No evidence of injury Risk of long-term events
		Stage 0B	Return to baseline values	Ongoing kidney damage/injury Loss of renal reserve
		Stage 0C	Increase < 1.5-fold	Ongoing kidney damage/injury
		Stage 1	1.5-fold increase	Ongoing kidney damage/injury
		Stage 2	2-fold increase	Ongoing kidney damage/injury
		Stage 3	3-fold increase	Ongoing kidney damage/injury

## Data Availability

The data are available from the corresponding author on reasonable request.
